# WISE-Therapy (What’s Important: Schedule and Engage) and bouldering psychotherapy for depression: A randomized clinical trial

**DOI:** 10.1186/s12916-026-04918-5

**Published:** 2026-05-27

**Authors:** Johannes Kornhuber, Piotr Lewczuk, Eva-Maria Siegmann, Sophia Schneider, Lola Eversmann, Leona Kind, Katharina Luttenberger

**Affiliations:** 1 Department of Psychiatry and Psychotherapy, Friedrich-Alexander-Universität Erlangen-Nürnberg (FAU), Schwabachanlage 6, 91054 Erlangen, Germany; 2 Department of Neurodegeneration Diagnostics, Medical University of Białystok, and Department of Biochemical Diagnostics, University Hospital of Białystok, Białystok, Poland; 3 Section of Medical Psychology and Medical Sociology, Department of Psychiatry and Psychotherapy, Uniklinikum Erlangen, Friedrich-Alexander-Universität Erlangen-Nürnberg (FAU), Schwabachanlage 6, 91054 Erlangen, Germany; 4 University for Continuing Education Krems (Donau-University Krems), Faculty for Health and Medicine, Department for Psychosomatic Medicine and Psychotherapy, Dr.-Karl-Dorrek-Straße 30, 3500 Krems an der Donau, Austria

**Keywords:** Depression, WISE-Therapy (WISE-T), Bouldering Psychotherapy (BPT), affective disorder, climbing, cognitive bias, group psychotherapy, physical activity, decision-making, Eisenhower Principle

## Abstract

**Background:**

Group therapies address the growing treatment demand-supply gap for depression. This study evaluated whether the novel WISE-Therapy (WISE-T; What’s Important: Schedule and Engage) and Bouldering Psychotherapy (BPT) are more effective in reducing depressive symptoms in an outpatient group therapy setting than treatment as usual (TAU).

**Methods:**

This randomized, controlled, assessor-blinded, longitudinal, clinical trial was conducted in a naturalistic monocentric setting in Erlangen (Germany) between April 2022 and March 2023, with follow-up until July 2024. 128 adults were randomly assigned to WISE-T (44), BPT (40), or TAU (44). In 10 weekly 2-hour group sessions, WISE-T focused on identifying what is important, scheduling meaningful activities, and engaging in them for long-term mental health, and BPT combined psychotherapy with bouldering, while the control group received TAU. Depression severity was measured with the Montgomery-Asberg Depression Rating Scale at baseline, post-intervention, and one-year follow-up, and analyzed with a hybrid marginal model.

**Results:**

Both intervention groups showed significantly greater reduction in depressive symptoms than controls at post-intervention (WISE-T: -5.5 points, 95%-CI [-9.5 to -1.5], P = .007, d = 0.62; BPT: -4.2 points, 95%-CI [-8.2 to -0.3], P = .037, d = 0.48). Response rates were 35.3% for WISE-T, 29.7% for BPT, and 21.1% for controls. Remission rates were significantly higher in WISE-T (17.6%) than in controls (2.6%, P = .047). Treatment adherence was good with minimal adverse events reported. The secondary outcomes showed significant results for anxiety, sense of coherence, self-efficacy, and mindfulness during physical activity.

**Conclusions:**

Both interventions demonstrated efficacy in reducing depressive symptoms compared with controls in this single-center setting. The study supports the integration of both therapies into outpatient care settings.

**Trial registration:**

Study procedures received approval from the Friedrich-Alexander-Universität Erlangen-Nürnberg Ethics Committee (Ref. 21-332-B) on December 13, 2021. The Trial was preregistered in March 2022 via ISRCTN12347878.

## Background

Major Depressive Disorder (MDD) is one of the most prevalent and burdensome mental health conditions in the world [1]. Despite the availability of evidence-based psychotherapies, significant treatment gaps persist due to limited accessibility, long waiting times, and insufficient therapeutic resources [2–5]. Group therapy formats offer a promising approach to address this growing demand by providing effective treatment to more individuals simultaneously. However, there is a need for innovative, short-term group interventions that are both clinically effective and appealing to patients. To address this gap, we developed two novel group therapies for outpatients with depression: WISE-Therapy (WISE-T; What’s Important: Schedule and Engage) and Bouldering Psychotherapy (BPT).

WISE-T is a novel psychotherapeutic intervention developed to address core challenges associated with MDD, including anhedonia, cognitive distortions, rumination, impaired decision-making, loss of interest, and social isolation. The therapy’s name encapsulates its three core components: identifying what is important in one’s life, scheduling these activities through concrete planning and habit formation, and actively engaging in them — thereby counteracting the avoidance and withdrawal characteristic of depression. The therapeutic approach is grounded in the Eisenhower Matrix, a framework that categorizes activities on the basis of their importance and urgency [6]. Recent research has identified a phenomenon known as the “mere urgency effect,” where individuals tend to prioritize tasks with shorter completion windows over tasks with larger payoffs, even when the former are objectively less beneficial [7]. Activities that are important but not urgent —  such as regular exercise, maintaining relationships, engaging in personal development, and practicing mindfulness — contribute significantly to psychological well-being and life satisfaction, yet they are often postponed in favor of more pressing tasks. Depression inherently limits cognitive, emotional, and physical resources. A key feature of WISE-T is how it addresses this fundamental constraint: by teaching patients to focus on what is truly important, it prevents the dissipation of already scarce resources on less meaningful tasks. WISE-T aims to help individuals recognize and overcome the mere urgency effect, shifting their focus toward important activities that can improve overall well-being and alleviate depressive symptoms. While building on cognitive-behavioral therapy, WISE-T places particular emphasis on long-term decision-making and life planning. At the same time, WISE-T is based on a simple principle that considers the limited resources of patients with depression.

BPT is an innovative, body-oriented treatment for depression that combines cognitive-behavioral therapy (CBT) with the physical and mental challenges of bouldering, a rope-free form of low-height climbing on rocks or artificial walls. It is designed as form of psychotherapy enhanced with physical exercise, which enables patients to experience psychological concepts (e.g. fear exposure) during therapy sessions rather than a mere physical exercise. Bouldering was selected for its unique therapeutic properties and the compatibility with group treatment: it requires focused attention and problem-solving (each climbing route presents a specific challenge), involves manageable risk-taking and mastery experiences, facilitates social interaction through the group setting, and provides natural metaphors for overcoming life obstacles — features that distinguish it from endurance-based activities such as running. Additionally, patients with very different levels of fitness can participate together in one group side by side. Meta-analytic evidence indicates that physical activity exerts a significant antidepressant effect [8–11], which is reflected in its recommendation in the German national clinical practice guidelines for the treatment of unipolar depression [12]. The distinctive feature of BPT lies in its integration of two empirically validated core treatments for depression: psychotherapy and physical activity. An initial randomized trial showed that BPT significantly reduced depressive symptoms compared with a waitlist control group, with benefits lasting for at least one year [13, 14]. A recent large-scale multi-center trial found that BPT was more effective in treating depression than physical exercise alone and was non-inferior to cognitive-behavioral group therapy, while also being cost-effective [15–17]. BPT’s therapeutic mechanisms include increased self-efficacy, improved body awareness, social support, and using climbing challenges as metaphors for life situations [18].

The primary outcome measure was the Montgomery-Asberg Depression Rating Scale (MADRS) [19]. To investigate potential mechanisms of action and differential effects of the interventions, we included several secondary outcomes that assessed different clinical domains relevant to depression treatment: Anxiety frequently co-occurs with depression, and previous studies on BPT have shown significant effects on anxiety reduction. Self-efficacy was assessed, as it has been identified as a potential mechanism of action in BPT [18], and WISE-T aims to improve mastery experiences through better decision-making. Both interventions aim to enhance participants’ sense of coherence — WISE-T through improved prioritization and decision-making frameworks and BPT through embodied experiences. Additionally, the interventions were expected to alter participants’ internal and external locus of control — WISE-T through better understanding of controllable versus uncontrollable factors and BPT through mastery experiences in climbing. To examine whether WISE-T might enhance body awareness through improved attention regulation and assess the body-focused mechanisms of BPT, we measured mindfulness during physical activity. Finally, it has been proposed that a key mechanism of WISE-T is that it enhances participants’ ability to engage in deliberate cognitive processing as opposed to relying on automatic responses. We evaluated this cognitive processing with a cognitive reflection test. 

In this study, we evaluated two innovative group therapies for depression compared with a control group (CG) that received treatment as usual (TAU). We hypothesized that participants in both intervention groups (IGs) would experience a significantly greater reduction in depressive symptoms compared with the CG. This design enabled us not only to test the efficacy of these novel approaches but also to potentially expand the range of evidence-based treatment options available to patients with depression.

## Methods

### Study design

For a detailed description of the study design, please see our design paper [20]. Deviating from the study protocol, the following changes were made: First, WISE-Therapy (What’s Important: Schedule and Engage) was initially named “Mental Model Therapy” (MMT). The therapy was renamed to better reflect its core therapeutic approach: identifying what is important, scheduling these activities, and engaging in them. Second, changes were made to the statistical analysis (as specified in the Sample Size and Statistical Analysis section). Third, regarding the secondary outcomes, no specific analyses were prespecified; these were therefore treated as exploratory, and the analyses conducted are first described here in the Sample Size and Statistical Analysis section.

The study was designed as a randomized, controlled, interventional, prospective, parallel, longitudinal, assessor-blinded trial, comprising three arms (WISE-T, BPT, CG). The trial commenced on October 1, 2021, with recruitment running from March 29, 2022, to February 28, 2023, in the Erlangen/Nuremberg/Fürth metropolitan area in Germany.

The study was conducted in four consecutive waves. Data collection for the primary outcome (depression) was based on structured video interviews at baseline, prior to the initiation of therapy (t0), immediately following the intervention (t1), and at the 12-month post-intervention follow-up (t2). The follow-up was completed in July 2024.

A detailed overview of all subsequent assessments and their corresponding time points is presented in our study protocol [20].

Study clinicians at headquarters and the group therapists were responsible for safety monitoring, including assessment of adverse events (AEs). Study procedures received approval from the Friedrich-Alexander-Universität Erlangen-Nürnberg Ethics Committee (Ref. 21-332-B) on December 13, 2021. The Trial was preregistered via ISRCTN12347878.

### Participants

Several recruitment strategies (press releases; informative events; flyers and posters distributed at psychiatric hospitals, psychotherapist offices, pharmacies, health insurance companies and at other psychological services; homepage, Instagram & Facebook accounts) were used to ensure the highest possible representativity of the sample. Individuals opting to participate were required to undergo a computer-assisted diagnostic video screening interview to assess inclusion and exclusion criteria, and to furnish written informed consent. Inclusion criteria [20] included meeting the diagnostic criteria for an acute episode of major depression (single or recurrent) according to the DSM-5, using a standardized diagnostic screening interview [21]. Briefly, exclusion criteria comprised being < 18 years of age, having a Body Mass Index (BMI) < 17.5 or > 40, current participation in another group psychotherapy, initiation or change of psychiatric medications within 8 weeks, planning an inpatient stay during the intervention period, having physical conditions contraindicating bouldering, exhibiting specific severe psychiatric disorders (psychosis/manic episode within the last 5 years, substance addiction with recent substance abuse, self-harming behavior within the past year, or current organic diseases with psychiatric symptoms like dementia or delirium), or experiencing acute suicidality.

### Randomization and masking

Blockwise randomization was conducted externally by the Institute of Medical Informatics, Biometry, and Epidemiology (IMBE) at Friedrich-Alexander-Universität Erlangen-Nürnberg. Only patient codes, age, sex, and treatment preferences were used in a stochastic minimization algorithm to reduce group imbalances [22]. Interviewers assessing the study outcomes remained blind to the individual randomization and reminded participants to keep their treatment condition confidential.

### Interventions

Both interventions were delivered in groups of about 10 participants at either the Psychiatric Unit of University Hospital Erlangen (WISE-T) or a local bouldering gym (BPT). They included 10 weekly, 2-hour sessions facilitated by two therapists for each intervention — two trained in manualized WISE-T and two trained in manualized BPT before initiation of the intervention at the headquarter. All therapists were qualified psychiatrists, psychotherapists, or psychologists with extensive experience in treating depression. A structured manual ensured consistency across all sessions. Both WISE-T and BPT are delivered in a group setting, leveraging social support and shared learning experiences.

#### WISE-Therapy (WISE-T)

WISE-Therapy is based on the Eisenhower Principle, which guides participants to identify what is truly important, schedule long-term meaningful activities, and actively engage in them rather than defaulting to urgent but less important tasks. Key therapeutic elements (shown in Table 1; Additional file 1; Additional file 2) include: 1. Value clarification: Patients identify core values to guide the selection of important activities. 2. Positive habit formation: Therapy fosters stable habits aligned with identified values to schedule and engage in these activities. 3. Cognitive restructuring: WISE-T addresses common cognitive biases and decision-making errors identified in behavioral economics, such as the sunk cost fallacy, hindsight bias, and the focusing illusion to help patients make more rational choices. 4. Practical tools: strategies such as the Ivy Lee method support prioritization and time management. The WISE-T manual will be available via www.WISE-Therapie.de.

**Table 1:** Overview Therapy Sessions BPT and WISE-T

Session	WISE-T	BPT
1	Introduction and Eisenhower Principle and Value Clarification	Introduction to Bouldering and Mindfulness
2	Habits and Pareto Principle	Body Awareness and the Body’s Center of Gravity
3	Balance Model and Ivy-Lee Method	Dealing with Boundaries
4	Solomon Paradox and Focusing Illusion	Expectations and Aspirations
5	Saying No and Clear Thinking	Self-Efficacy, Achievement, and Pride
6	Active Language and Confirmation Bias	Self-Worth
7	Value Clarification and Sunk Cost Fallacy	Fear and Confidence I
8	Hindsight Bias and Decision-Making Strategies	Fear and Confidence II
9	Radical Acceptance and Occam’s Razor	Social Relationships
10	Closing: Reflection and Transfer to Everyday Life and Farewell	Closing: Problem Solving, Reflecting on Lessons Learned, Transfer to Everyday Life, and Farewell

*BPT* Bouldering Psychotherapy, *WISE-T* WISE-Therapy

#### Bouldering psychotherapy (BPT)

The bouldering intervention integrates psychotherapy and bouldering, a special form of climbing to moderate heights (up to about 3 meters) without the use of ropes or harnesses, with potential falls or jumps cushioned by thick mats. Sessions address psychological topics relevant to the development and maintenance of depression (shown in Table 1) and follow a standardized BPT manual (www.boulderpsychotherapie.de). Each session consists of 3 parts: The introduction phase (20 minutes) was conducted in a separate room and began with a mindfulness exercise, followed by a presentation of the session’s topic and related psychoeducation. During the action phase (75 minutes), participants completed topic-related bouldering exercises in the bouldering hall, designed to evoke emotions, uncover characteristic patterns, and facilitate new experiences. Each session concluded with a relaxation exercise and a group discussion about the lessons that were learned and how to transfer them to daily life (25 minutes).

#### Control group (CG)

The Control Group (CG) represented treatment as usual (TAU) within the German healthcare system, without study-specific therapy. Participants could access standard psychotherapeutic or psychiatric treatments and were incentivized with vouchers or bouldering passes. Additionally, they were offered priority admission to outpatient BPT sessions after the study.

### Assessment procedures and data quality management

Data collection was carried out using a combination of self-report questionnaires and standardized video conference interviews. Scheduling and conducting the interviews was facilitated through the certified software *Samedi*. The interviews were conducted by university students enrolled either in clinical psychology or medicine, who had been trained in advance at the headquarter. Participants were instructed to complete the self-report questionnaires before their scheduled video interviews. All data were recorded using the web-based data collection platform *REDCap2*. Interviewers and therapists were supervised by staff members of the study center and regularly discussed therapeutic progress and any emerging uncertainties in scheduled supervision meetings. Participants therapy attendance and adverse events were documented during each intervention session.

### Outcome

#### Primary outcome

The primary outcome measure was the Montgomery-Asberg Depression Rating Scale (MADRS) [19], which employs the Structured Interview Guide for the Montgomery-Asberg Depression Rating Scale (SIGMA) [23].

There are different MADRS cut-off scores, which define *remission* as ranging from scores of 12 or less to 7 or less [24–26]. Similar to our recent remission analysis [27] and in accordance with Riedel et al. [25], we chose the most conservative definition of remission, defining participants as remitted if they reached a value of 7 or less on the MADRS. *Response* was defined as a reduction of at least 46% from the pre-therapy (t0) MADRS value to the post-therapy (t1) value as suggested by Riedel et al. [25]. Clinically, a reduction in MADRS scores serves as an indicator of *treatment efficacy*. As we defined in recent studies[28], half an average severity interval ( < 10 remission, 10 to 20 mild depression, 21 to 31 moderate depression [26, 29]) appears clinically relevant, which leads to a clinically relevant reduction of at least 5 points on the MADRS scale.

#### Secondary outcomes

The Generalized Anxiety Disorder Scale-7 (GAD-7) is a brief self-report questionnaire used to assess anxiety levels. It consists of seven items, each rated on a 4-point scale ranging from 0 (*not at all*) to 3 (*nearly every day*) [30]. Total scores range from 0 to 21, with higher scores indicating more severe anxiety symptoms: 5 to 9 for mild anxiety, 10 to 14 for moderate anxiety, and ≥ 15 for severe anxiety [30].

The Self-Efficacy Scale (SWE) measures an individual’s belief in their ability to successfully cope with difficult situations [31, 32]. The scale includes 10 items, rated on a 4-point scale ranging from 1 (*not true*) to 4 (*exactly true*), with total scores ranging from 10 to 40, where higher scores indicate greater self-efficacy [31, 32].

The Sense of Coherence Scale (SOC) measures a person’s sense of coherence, which refers to having a sense of confidence that they are able to make reasonable and probable predictions about developments in their internal and external environment [33, 34]. The short nine-item version of the SOC (SOC-L9) was used, with items rated on a 7-point scale, and scores ranging from 9 to 63. Higher scores indicate a stronger sense of coherence.

The Internal-External Locus of Control Scale (IE-4) assesses a person’s beliefs about the degree of control they have over events in their life [35]. The scale includes two subscales: internal and external locus of control, each with two items rated on a 5-point scale ranging from 1 (*not at all true*) to 5 (*completely true*). Scores are averaged for each subscale, with higher scores indicating a stronger belief in internal or external control.

The State Mindfulness Scale for Physical Activity (SMS-PA-12) [36, 37] measures state mindfulness of the mind and body and was developed to measure mindfulness during physical activity [38]. In this analysis, we used the overall score, consisting of 12 items, each rated on a 5-point scale ranging from 0 (*not at all*) to 4 (*very much*). Higher scores indicate higher mindfulness [38]. We used the validated German version of the SMS-PA, the SMS-PA-G [39].

The Cognitive Reflection Test (CRT-3) evaluates cognitive processing by assessing the tendency to reflect before responding to heuristic-based questions [40, 41]. The test consists of three questions designed to differentiate between reflective and intuitive thinking.

### Sample size and statistical analysis

The sample size was determined on the basis of data from previous studies involving BPT; for details, please see our design paper [20]. Originally, the analysis was planned using a mixed-methods ANOVA. The a priori calculated minimum sample size for the ANOVA could not be reached due to missing data at t1 (109 complete cases vs. the required 114). This may have resulted in insufficient statistical power for conducting the originally planned mixed-methods ANOVA. The main reason to switch from ANOVA-like estimations to regression models was estimators asymptotic consistency offered by the later. Therefore, all models were calculated by maximization of the likelihood function. In contrast to somehow old-fashioned ANOVA family (incl. ANCOVA, MANOVA, etc.), the modern regression methods — as long as they rely on the likelihood theory — are asymptotically consistent in presence of missingness, assuming that the data are Missing At Random (MAR). This is due to the property of the likelihood estimation called ignorability [42–45]. Indeed, drop outs may also result in lowering power and inflated p-values of the model estimates as well as the post-estimation contrasts, which we hereby acknowledge. Additional there were critical violations of statistical assumptions of the ANOVA — specifically, the lack of homogeneity of error variances — also reasoning an alternative analytical approach. The data analyses were conducted using Stata V.17.0 [46] and IBM SPSS Statistics V.28 [47]. With three measurement points for each participant, the study represents a longitudinal design with measurement occasions (level-1) nested within the participants (level-2). This design calls for a modeling method that takes the conditional correlations of the outcomes into consideration. It can be achieved by a (random intercept) Linear Mixed Model (LMM) or — derived from there — a marginal model operating on a properly structured residual covariance matrix. An alternative method is a hybrid marginal model (HMM) that combines the properties of the two formulations. This model, in order to account for correlated residuals when the measurement occasions are unevenly spaced, comprises a random intercept and an exponential structure of the covariance matrix that reflects the serial correlations of the level-1 residuals that do not approach zero as the time lag increases [48]. Nested models with different explanatory variables (EVs) like age, sex, BMI, additional psychotherapy etc. were compared with one another using the Likelihood Ratio Test (LRT). After the best-fitting model (among M1, M2, M3 and M4) was chosen, marginal contrasts were post-estimated to find differences in the outcome between the three treatment arms at the three occasions with the treatment effect represented by the occasions-by-groups interaction. Standardized effect sizes were estimated by dividing model-estimated contrasts by the total standard deviation, computed as the square root of the sum of variance components (between-subject variance + residual variance), following recommendations for effect size computation in mixed models [49]. The procedure for the secondary outcomes GAD-7, SWE, SOC, and SMS-PA was analogous to the primary outcome. The categorical outcome variable CRT was modeled with ordinal logistic regression. The subscales of IE-4 were analyzed descriptively. All analyses followed the intention-to-treat (ITT) principle: all randomized participants were analyzed according to their original group assignment, irrespective of treatment adherence or completion of assessments. For all hypothesis testing we used all available data; P < .05 was considered significant.

## Results

### Descriptive outcomes

**Fig. 1:**
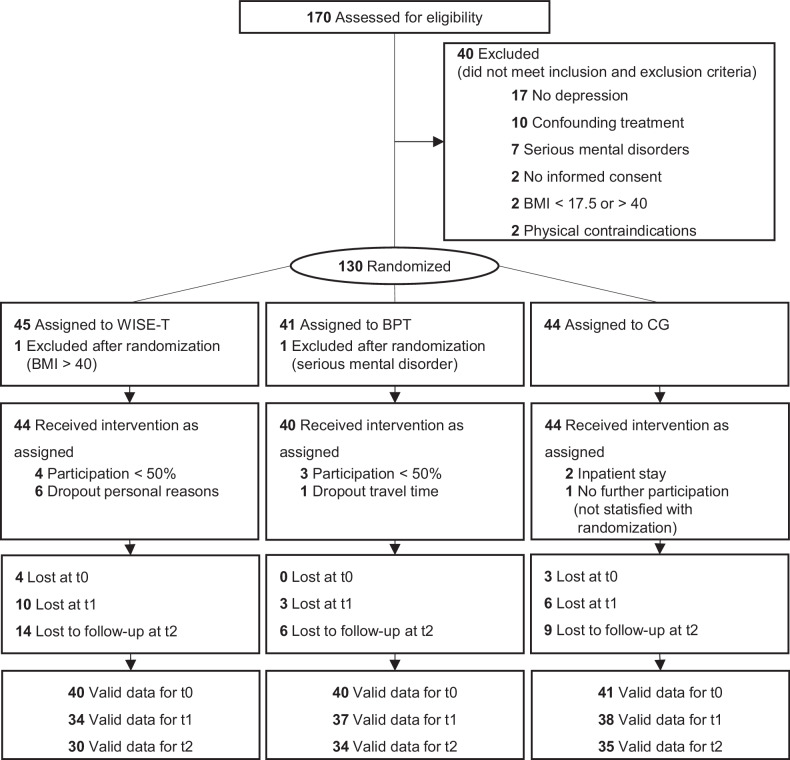
CONSORT Diagram. BMI, Body Mass Index; BPT, Bouldering Psychotherapy; CG, Control Group; WISE-T, WISE-Therapy; t0, pretest; t1, posttest; t2, one year follow-up

Between April 2022 and March 2023, 170 individuals were initially recruited, with 128 meeting the inclusion criteria. Participants were randomly assigned to one of three groups: 40 to BPT, 44 to WISE-T, and 44 to the CG (shown in Fig. 1). After the intervention, 19 patients (14.8%) were unavailable for assessment, and 29 patients (22.7%) were missing at follow-up. A chi-square test showed no significant difference in dropout rates between groups, χ²(2) = 3.33, P = .189. Before randomization, 102 (79.7%) participants rated BPT as their preferred group allocation, followed by 25 (19.5%) participants who preferred WISE-T, and 1 (0.8%) person who preferred TAU. Through stratified randomization, these preferences were evenly distributed across the groups with a match between treatment preference and group allocation in 41 cases (32%).

With the exception of differences in comorbidities, no significant differences were observed between the groups at baseline (shown in Table 2). Approximately half of the participants had been experiencing their current depressive episode for a duration between six months and two years. One third of the sample had experienced one to two depressive episodes in total, another third reported three to four episodes, and the remaining third had experienced five or more episodes or suffered from chronic depression lasting longer than two years. Additionally, around 38% of participants were receiving concurrent psychotherapy, and 57% were taking antidepressant medication.

**Table 2:** Baseline Characteristics

Variable	WISE-T (n = 44)	BPT (n = 40)	CG (n = 44)	Total (n = 128)
**Age**, mean (SD)	38.6 (13.7)	40.9 (14.8)	39.9 (15.1)	39.8 (14.5)
**Sex**, No. (%)				
Female	27 (61.4)	25 (62.5)	28 (63.6)	80 (62.5)
Male	17 (38.6)	15 (37.5)	16 (36.4)	48 (37.5)
**BMI**, mean (SD), kg/m²	25.1 (5.9)	25.9 (4.9)	24.5 (4.4)	25.1 (5.1)
**Family status**, No.^a^ (%)				
Single	21 (51.2)	21 (52.5)	26 (60.5)	68 (53.1)
Married/living in a partnership	17 (41.5)	12 (30.0)	10 (23.3)	39 (30.5)
Separated/divorced/widowed	3 (7.3)	7 (17.5)	7 (16.3)	17 (13.3)
**School education**, No.^a^ (%)				
< 9 years (not yet completed school)	0 (0)	0 (0)	2 (4.7)	2 (1.6)
9 years (German Hauptschulabschluss/Secondary School)	0 (0)	4 (10.0)	2 (4.7)	6 (4.7)
10 years (German Mittlere Reife/Vocational School)	7 (17.1)	7 (17.5)	11 (25.6)	25 (19.5)
> 10 years (German Hochschulabschluss/Grammar School)	34 (82.9)	29 (72.5)	28 (65.1)	91 (71.1)
**Current occupation: yes**, No.^a^ (%)	32 (78.0)	29 (72.5)	32 (74.4)	93 (72.7)
**Number of depression criteria met**, mean (SD)	6.9 (1.2)	6.6 (1.1)	6.9 (1.1)	6.8 (1.2)
**Duration of the current depressive episode**				
< ½ year	12 (27.3)	6 (15.0)	12 (27.3)	30 (23.4)
½ year - 2 years	20 (45.5)	21 (52.5)	18 (40.9)	59 (46.1)
> 2 years	12 (27.3)	13 (32.5)	14 (31.8)	39 (30.5)
**Current or past psychiatric comorbidities: yes**, No. (%)	8 (18.2)	6 (15.0)	17 (38.6)	31 (24.2)
**Number of depressive episodes**, No. (%)				
1 - 2	15 (34.1)	10 (25.0)	18 (40.9)	43 (33.6)
3 - 4	18 (40.9)	15 (37.5)	9 (20.5)	42 (32.8)
≥ 5 or chronic depression ( > 2 years)	11 (25.0)	15 (37.5)	17 (38.6)	43 (33.6)
**Additional psychotherapy: yes**, No.^b^ (%)	16 (40.0)	21 (52.5)	12 (29.3)	49 (38.3)
**Antidepressant medication: yes**, No. (%)	24 (54.5)	23 (57.5)	26 (59.1)	73 (57.0)
Antidepressants: yes, No. (%)	22 (50.0)	21 (52.5)	25 (56.8)	68 (53.1)
Plant-derived antidepressants: yes, No. (%)	2 (4.5)	1 (2.5)	0 (0)	3 (2.3)
Neuroleptics: yes, No. (%)	1 (2.3)	4 (10.0)	5 (11.4)	10 (7.8)
Thyroid medication: yes, No. (%)	1 (2.3)	0 (0)	0 (0)	1 (0.8)
Phase prophylactics: yes, No. (%)	0 (0)	0 (0)	0 (0)	0 (0)
Others with presumed antidepressant effect: yes, No (%)	1 (2.3)	1 (2.5)	2 (4.5)	4 (3.1)
**Past psychotherapeutic treatments**, No.^b^ (%)				
0	17 (42.5)	15 (37.5)	11 (26.8)	43 (33.6)
1	11 (27.5)	6 (15.0)	11 (26.8)	28 (21.9)
> 1	12 (30.0)	19 (47.5)	19 (46.3)	50 (39.1)
**Number of therapy attempts in the past**, No.^c^ (%)				
0	3 (7.5)	6 (15.0)	6 (15.0)	15 (11.7)
1	11 (27.5)	8 (20.0)	8 (20.0)	27 (21.1)
2 - 3	15 (37.5)	16 (40.0)	12 (30.0)	43 (33.6)
> 3	11 (27.5)	10 (25.0)	14 (35.0)	35 (27.3)
**Preferred group assignment**, No. (%)				
BPT	36 (81.8)	32 (80.0)	34 (77.3)	102 (79.7)
WISE-T	8 (18.2)	8 (20.0)	9 (20.5)	25 (19.5)
CG	0 (0)	0 (0)	1 (2.3)	1 (0.8)

*BMI* measured Body Mass Index, *BPT* Bouldering Psychotherapy, *CG* Control Group, *WISE-T* WISE-Therapy

a n WISE-T: 41, n CG: 43, n total: 124 (4 missing values)

b n WISE-T: 40, n CG: 41, n total: 121 (7 missing values)

c n WISE-T: 40, n CG: 40, n total: 120 (8 missing values)

On average, participants attended 7.0 WISE-T sessions (SD = 3.7) or 8.2 BPT sessions (SD = 2.4). Among the CG participants, 10 individuals (23%) engaged in BPT during the year following the initial treatment period. At the t1 interview, 11 participants (25%) in the WISE-T group reported experiencing significant incidents, such as injuries, accidents, psychological crises, or hospitalizations, during the past 10 weeks. Among these incidents, one individual indicated that the incident was therapy-related, specifically noting that an issue arising from therapy had preoccupied them for an extended period, categorizing it as a mild adverse event. Similarly, in the BPT group, 10 participants (25%) reported comparable incidents. Of these incidents, two individuals identified incidents directly linked to the therapy: one involved a ligament tear while bouldering (moderate adverse event), whereas the other involved the re-emergence of a traumatic memory from the past (mild adverse event). Additionally, the therapists reported a minor adverse event that involved an abdominal abrasion in the BPT group. In the CG, 12 participants (27%) reported incidents, a proportion comparably high to that of the IGs. One-year post-therapy, 17 individuals (13%) reported significant incidents during the preceding 12 months, distributed across the groups as follows: 4 in the WISE-T group, 6 in the BPT group, and 7 in the CG. Among these incidents, one injury (shoulder surgery) was associated with a fall while privately bouldering.

### Primary outcome

16 (12%) of the t0 and 15 (11%) of the t1 MADRS surveys were evaluated by different pairs of raters. The interrater reliabilities for the MADRS assessed with the SIGMA, were found to be good to very good (ICC = .921, 95%-CI [.829 to .963], *P* < .001, *n* = 31) at both t0 (ICC = .780, 95%-CI [.369 to .923], *P* = .001, *n* = 16) and t1 (ICC = .962, 95%-CI [.890 to .987], *P* < .001, *n* = 15).

The internal consistency of the 10-items of the MADRS was examined using Cronbach’s alpha. The analysis yielded values of .79 at t0 and .88 at both subsequent time points t1 and t2, indicating acceptable to high internal consistency. These reliability estimates align with those reported in prior research [50, 51].

**Fig. 2:**
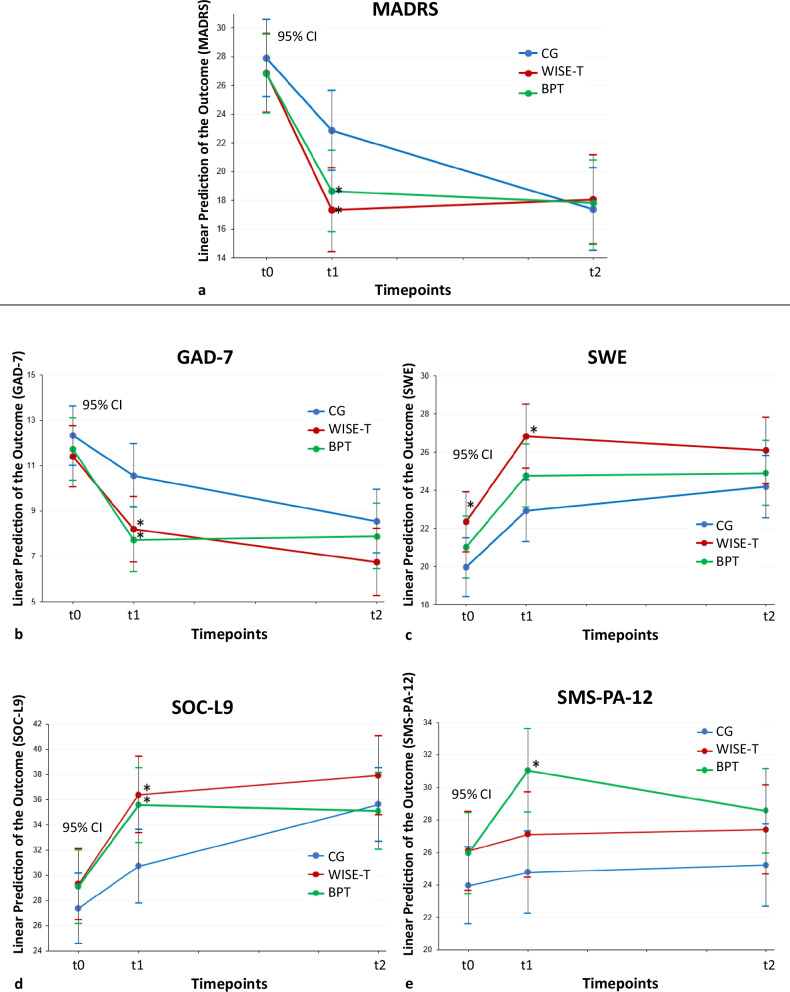
Time evolution of the marginal linear predictors of the outcome variables in the three groups of patients post-estimated from M1. **a**, Primary Outcome; **b**-**e**, Secondary Outcomes; BPT, Bouldering Psychotherapy; CG, Control Group; WISE-T, WISE-Therapy; GAD, Generalized Anxiety Disorder Scale-7; MADRS, Montgomery-Asberg Depression Rating Scale; SMS-PA-12, State Mindfulness Scale for Physical Activity; SOC-L9, Sense of Coherence Scale; SWE, Self-Efficacy Scale; t0, pretest; t1, posttest; t2, one year follow-up; 95%-CI, 95% Confidence Interval. Asterisks denote significant (at the 0.05 level) differences in the contrasts compared with the CG

In the model building, HMM and marginal models with an unstructured covariance matrix (MM) with an increasing number of explanatory variables were considered (shown in Table 3) and compared with one another using Likelihood Ratio Tests. The contrasts were post estimated after models controlling for age, sex, BMI, antidepressant medication and additional psychotherapy, yielding comparable significant contrasts to those observed in M1. Although MM (e.g. M2) demonstrated better statistical performance than HMM (e.g. M1), we decided to use M1 for the post-estimation, as we believe that its covariance structure reflects the study design better. Using M1, at t1, both IGs’ post-estimate marginal contrasts showed significantly lower MADRS scores compared with the CG (shown in Fig. 2. a). In the WISE-T, the difference was approximately -5.5 points in comparison with CG, P = .007 (SE = 2.1, 95%-CI [-9.6 to -1.5]), and in the BPT, it was approximately -4.2 points, P = .037 (SE = 2.0, 95%-CI [-8.2 to -0.3]). Standardized effect sizes at post-treatment were d = 0.62 for WISE-Therapy versus CG and d = 0.48 for BPT versus CG, representing medium and small-to-medium effects, respectively. There were no significant differences between the groups at t0 and t2.

**Table 3:** Contrasts from M1, M2, and M3

		M1 (HMM) time, group, time x group		M2 (MM unstructured) time, group, time x group		M3 (HMM) M1 + age, sex, BMI, additional psychotherapy	
**Groups**	**Time**	**Contrast (SE) [95%-CI]**	***P *Value**	**Contrast (SE) [95%-CI]**	***P *Value**	**Contrast (SE) [95%-CI]**	***P *Value**
WISE-T vs. CG	t0	-1.03 (1.96) [-4.87 to 2.81]	.600	-1.03 (1.66)[-4.29 to 2.23]	.536	-1.12 (1.95)[-4.94 to 2.70]	.566
	t1	-5.51 (2.06) [-9.55 to -1.47]	**.007**	-5.30 (2.15) [-9.51 to -1.09]	**.014**	-5.61 (2.05) [-9.63 to -1.59]	**.006**
	t2	0.68 (2.16) [-3.56 to 4.92]	.753	1.03 (2.43) [-3.74 to 5.79]	.672	0.59 (2.16) [-3.64 to 4.81]	.785
BPT vs. CG	t0	-1.05 (1.96) [-4.89 to 2.79]	.591	-1.05 (1.66) [-4.31 to 2.21]	.526	-1.37 (1.97) [-5.23 to 2.49]	.486
	t1	-4.22 (2.02) [-8.19 to -0.26]	**.037**	-4.19 (2.11) [-8.33 to -0.05]	**.047**	-4.55 (2.03) [-8.53 to -0.57]	**.025**
	t2	0.47 (2.10) [-3.64 to 4.59]	.822	0.58 (2.36) [-4.05 to 5.21]	.806	0.17 (2.11) [-3.97 to 4.31]	.936

*BMI* measured Body Mass Index, *BPT* Bouldering Psychotherapy, *CG* control group, *HMM* Hybrid Marginal Model with exponential covariance matrix, *MM* unstructured, Marginal Model with unstructured covariance matrix, *WISE-T* WISE-Therapy, *SE* standard error, *t0* pretest, *t1* posttest, *t2* one year follow-up, 95%-CI, 95% Confidence Interval

On a horizontal level, adjusted predictors of MADRS depression scores in the WISE-T group decreased from t0 to t1 by -9.6 points from 26.9 (SE = 1.4, 95%-CI [24.1 to 29.6]) to 17.3 (SE = 1.5, 95%-CI [14.4 to 20.3]) and to 18.0 (SE = 1.6, 95%-CI [14.9 to 21.1]) at follow-up. In the BPT group depression scores decreased by -8.2 points from 26.8 (SE = 1.4, 95%-CI [24.1 to 29.6]) at t0 to 18.6 (SE = 1.4, 95%-CI [15.8 to 21.4]) at t1 and 17.8 (SE = 1.5, 95%-CI [14.9 to 20.8]) at follow-up. The scores in the CG decreased by -5.1 points from 27.9 (SE = 1.4, 95%-CI [25.2 to 30.6]) at t0 to 22.8 (SE = 1.4, 95%-CI [20.1 to 25.6]) at t1 and 17.4 (SE = 1.5, 95%-CI [14.5 to 20.3]) at follow-up. A clinically relevant reduction of at least -5 points in depression scores was exceeded in all three groups.

Additionally, the proportion of response and remission at t1 were identified for patients with complete t0 and t1 MADRS data (nWISE-T = 34; nBPT = 37; nCG = 38). In the WISE-T group, 12 patients (35.3%) responded, with 6 (17.6%) of them going into remission. In the BPT group, 11 patients (29.7%) responded, and 3 (8.1%) went into remission; and in the CG, 8 patients (21.1%) responded, and 1 person (2.6%) went into remission. Comparing the IGs against the CG with χ^2^-tests, a significant difference in remission rate was found between WISE-T and the CG; exact two-sided Fisher test P = .047 (expected cell frequencies < 5), a small to moderate effect φ = 0.25.

### Secondary outcomes

Table 4 summarizes the contrasts from the best-fitting models for the GAD-7, SWE, SOC, and SMS-PA.

**Table 4:** Contrasts from the best fitting models for secondary outcomes

		GAD time, group, time x group		SWE time, group, time x group		SOC time, group, time x group		SMS-PA time, group, time x group	
**Groups**	**Time**	**Contrast (SE) [95%-CI]**	***P *Value**	**Contrast (SE) [95%-CI]**	***P *Value**	**Contrast (SE) [95%-CI]**	***P *Value**	**Contrast (SE) [95%-CI]**	***P *Value**
WISE-T vs. CG	t0	-0.91 (0.96) [-2.80 to 0.97]	.342	2.38 (1.13) [0.17 to 4.59]	**.035**	1.92 (2.02) [-2.04 to 5.88]	.342	2.14 (1.73) [-1.26 to 5.54]	0.217
	t1	-2.37 (1.02) [-4.38 to -0.36]	**.021**	3.90 (1.19) [1.57 to 6.22]	**.001**	5.65 (2.15) [1.44 to 9.87]	**.009**	2.33 (1.86) [-1.31 to 5.96]	0.209
	t2	-1.80 (1.04) [-3.85 to 0.24]	.083	1.89 (1.21) [-0.49 to 4.27]	.119	2.31 (2.18) [-1.97 to 6.59]	.290	2.18 (1.90) [-1.55 to 5.90]	0.252
BPT vs. CG	t0	-0.59 (0.97) [-2.50 to 1.31]	.542	1.07 (1.14) [-1.17 to 3.31]	.348	1.71 (2.05) [-2.30 to 5.72]	.403	2.00 (1.76) [-1.45 to 5.44]	0.256
	t1	-2.83 (1.01) [-4.81 to -0.84]	**.005**	1.84 (1.18) [-0.47 to 4.15]	.118	4.83 (2.13) [0.67 to 9.00]	**.023**	6.27 (1.83) [2.68 to 9.86]	**0.001**
	t2	-0.66 (1.03) [-2.67 to 1.35]	.521	0.72 (1.20) [-1.63 to 3.06]	.550	-0.51 (2.15) [-4.73 to 3.71]	.812	3.33 (1.86) [-0.31 to 6.98]	0.073

*BPT* Bouldering Psychotherapy, *CG* control group, *WISE-T* WISE-Therapy, *SE* standard error, *t0* pretest, *t1* posttest, *t2* one year follow-up, 95%-CI, 95% Confidence Interval

At t1, both IGs’ post-estimate marginal contrasts showed significantly lower GAD-7 scores compared with the CG (shown in Fig. 2. b). For WISE-T, the difference was approximately -2.4 points in comparison with the CG, P = .021 (SE = 1.0, 95%-CI [-4.4 to -0.4]), medium effect (d = 0.53), and for BPT, it was approximately -2.8 points, P = .005 (SE = 1.0, 95%-CI [-4.8 to -0.8]) medium effect (d = 0.63). There were no significant differences between the groups at t0 and t2.

Similarly, both IGs’ post-estimate marginal contrasts showed significantly higher SOC scores at t1 compared with the CG (shown in Fig. 2. d). In the WISE-T group, the difference was approximately 5.7 points in comparison with the CG, P = .009 (SE = 2.2, 95%-CI [1.4 to 9.9]), medium effect (d = 0.60), and in the BPT group, it was approximately 4.8 points, P = .023 (SE = 2.1, 95%-CI [0.7 to 9.0]), medium effect (d = 0.52), with no significant group differences at t0 and t2.

For the SMS-PA, only the BPT group’s post-estimate marginal contrasts showed significantly higher scores at t1 compared with the CG (shown in Fig. 2. e). The difference was approximately 6.3 points in comparison with the CG, *P* = .001 (SE = 1.8, 95%-CI [2.7 to 9.9]), medium-to-large effect (*d* = 0.78), with no significant group differences at t0 and t2.

The SWE showed significantly higher scores in the WISE-T group’s post-estimate marginal contrasts already at t0 and as well at t1 compared with the CG (shown in Fig. 2. c). At t0, the difference was approximately 2.4 points in comparison with the CG, P = .035 (SE = 1.1, 95%-CI [0.2 to 4.6]), small-to-medium effect (d = 0.46) and approximately 3.9, P = .001 (SE = 1.2, 95%-CI [1.6 to 6.2]), medium-to-large effect (d = 0.75) at t1.

Analogous to the Hybrid Marginal Model M3 of the primary outcome, HMM analyzes with additional explanatory variables (age, sex, BMI, medication, additional psychotherapy) were calculated in addition to the simple Hybrid Marginal Model M1 (time, group, time x group) for the secondary outcomes GAD, SWE, SOC, and SMS-PA. The results showed one additional significant difference in the BPT group’s post-estimate marginal contrast for the SWE score, which was 2.4 points higher than the CG at t1, *P* = .036 (SE = 1.2, 95%-CI [0.2 to 4.7]), with no significant group differences at t0 and t2.

The WISE-T group’s internal locus of control increased from 3.2 (SD = 0.8) at t0 to 3.8 (SD = 0.6) at t1 and 3.5 (SD = 0.8) at t2, whereas the external locus of control hardly decreased from 3.0 (SD = 0.9) at t0 to 2.9 (SD = 0.8) at t1 and 2.6 (SD = 1.0) at t2. The BPT group’s internal locus of control increased from 2.9 (SD = 0.7) at t0 to 3.3 (SD = 0.7) at t1 and 3.4 (SD = 0.6) at t2. The external locus of control decreased from 3.1 (SD = 0.8) at t0 to 2.7 (SD = 0.9) at t1 and 2.6 (SD = 0.7) at t2. In the CG, the internal locus of control barely changed from 3.1 (SD = 0.8) at t0 to 3.2 (SD = 0.8) at t1 and 3.4 (SD = 0.9) at t2. The external locus of control hardly decreased from 3.0 (SD = 0.8) at t0 to 2.9 (SD = 0.8) at t1 and 2.6 (SD = 0.8) at t2.

**Fig. 3:**
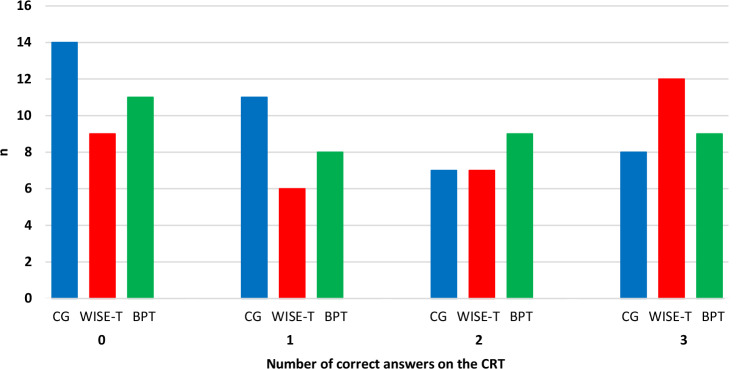
Number of correct answers to the Cognitive Reflection Task in the three groups. BPT, Bouldering Psychotherapy; CG, Control Group; CRT, Cognitive Reflection Task; WISE-T, WISE-Therapy; n, number of participants

Figure 3 presents the number of correct answers on the CRT for each group. The comparisons of marginal linear predictions showed no significant differences between the three groups, WISE-T compared with the CG: coefficient = 0.6, P = .128 (SE = 0.4, 95%-CI [-0.2 to 1.5]) and BPT compared with the CG: coefficient = 0.3, P = .450 (SE = 0.4, 95%-CI [-0.5 to 1.1]).

## Discussion

This randomized controlled trial showed that both WISE-T and BPT significantly reduced depressive symptoms compared with TAU. The effect sizes observed in our trial (d = 0.62 for WISE-T; d = 0.48 for BPT) are consistent with meta-analytic findings for psychotherapy of depression. A recent multiverse meta-analysis reported an average effect size of g = 0.56 (IQR 0.42–0.71) across 415 RCTs [52]. Our findings fall within this expected range, supporting the clinical relevance of both interventions. In the IGs, depression decreased by about one severity grade, from moderate severity pre-intervention to mild severity post-intervention, while the severity of depression in the CG remained moderate. The remission rates were notably higher in the WISE-T group compared with the CG. Although the CG also showed some improvement in depressive symptoms (MADRS reduction of 5.1 points), remission rates in the CG remained very low (2.6%), underscoring the added benefit of the structured interventions. It should be noted that while statistically significant, the absolute remission rates in the intervention groups were modest (17.6% for WISE-Therapy, 8.1% for BPT), reflecting the chronic and recurrent nature of the disorder in our sample.

Both interventions were group therapies that incorporated group cohesion and peer learning as significant factors, thereby influencing the efficacy of psychotherapies and addressing the growing treatment demand-supply gap [53, 54].

WISE-T introduces a unique mechanism for therapeutic change by addressing decision-making patterns that sustain depressive symptoms. Unlike traditional cognitive-behavioral approaches that target emotional responses to dysfunctional thoughts, WISE-T incorporates elements of behavioral economics, cognitive restructuring, and habit formation to promote more adaptive behavioral patterns. Whether these changes persist beyond the one-year follow-up period and how WISE-T compares directly with CBT remain important questions for future research.

WISE-T was effective despite 79.7% of participants expressing a preference for BPT. Our results are aligned with meta-analytic findings suggesting that treatment preference has no significant impact on clinical outcomes, although treatment preference may improve satisfaction and adherence [55].

This study adds to the growing body of support for the effectiveness of therapeutic climbing [56], with BPT showing similar reductions in depression severity as prior studies [15–17] despite a slightly lower response rate of 30% [27].

Regarding clinical relevance, notably, all three groups exceeded the predefined threshold of a reduction of at least 5 points on the MADRS scale, indicating a clinically meaningful reduction in depressive symptoms during the intervention period. The study was conducted during the lifting of COVID-19 restrictions (recruitment March 2022 – February 2023), a period associated with general improvements in population mental health as social reintegration occurred [57, 58]. Importantly, since this contextual factor affected all study arms equally, it does not bias the between-group comparisons. Additionally, hope — known to be inversely correlated with depression — may have influenced the CG, as participants of the CG knew they would likely be included in the hospitals´ BPT group after the end of the study [59]. The fact that both WISE-T and BPT demonstrated significant effects over TAU despite this favorable context underscores the robustness of the therapeutic effects.

At one-year follow-up, the between-group differences were no longer statistically significant. This convergence should not be interpreted as a loss of treatment efficacy. Rather, it reflects multiple concurrent processes: the intervention groups maintained their post-treatment improvements (WISE-T: MADRS 18.0; BPT: 17.8), while the control group showed continued gradual improvement consistent with the natural course of depression and ongoing treatment as usual, and partial crossover to BPT (see Results). These findings underscore the need for future research on maintenance strategies following acute group interventions.

The formal LRT showed better performance of the MM (M2) compared with the HMM (M1), though we think that the exponential structure of the covariance of the level-1 residuals in the HMM better reflected the study paradigm. Therefore, we eventually decided to use the HMM in a parsimonious setting with two fixed effects and their interaction in the mean structure and with the exponential covariance structure of the level-1 residuals.

Our secondary outcome analyses provided important insights into the mechanisms through which WISE-T and BPT reduce depressive symptoms, revealing both shared and intervention-specific pathways. Both interventions demonstrated significant improvements in anxiety symptoms at post-intervention compared with the CG. Similar to the depression scores, anxiety decreased by about one severity grade, from moderate severity pre-intervention to mild severity post-intervention in both IGs, while anxiety in the CG remained moderate. This finding aligns with the high comorbidity between depression and anxiety disorders and suggests that these group therapies address transdiagnostic processes. The reduction in anxiety from BPT aligns with previous findings on the anxiolytic effects of therapeutic climbing [15], while the similar improvement in WISE-T indicates that better decision-making and prioritization may also alleviate anxiety-related worries. Regarding self-efficacy, caution is warranted in interpreting the results. Although the WISE-T group showed significantly higher self-efficacy scores compared with the CG post-intervention, this difference was already present at baseline despite randomization. Thus, the post-intervention difference may reflect a continuation of pre-existing group characteristics rather than a treatment effect. The BPT group did not show significant differences in self-efficacy compared with the controls, which is somewhat unexpected given previous research identifying enhanced self-efficacy as a key mechanism in BPT [18]. This unanticipated result could be put into perspective by adding further explanatory variables to the model. When we controlled for age, sex, BMI, and additional treatment, we found the expected significant effect for the BPT group. The significant improvement in sense of coherence in both IGs highlights how these therapies help participants develop a more comprehensible, manageable, and meaningful understanding of their lives. WISE-T’s emphasis on structured prioritization and rational decision-making likely provides a cognitive framework that enhances coherence, while BPT’s embodied experiences and metaphorical climbing challenges may offer concrete demonstrations of how to overcome obstacles, both contributing to a stronger sense of coherence. As expected, state mindfulness during physical activity showed significant improvement only in the BPT group. This intervention-specific effect confirms BPT’s unique body-focused mechanism, leveraging the intense physical presence required in bouldering to enhance mindfulness. This mind-body integration presents a distinct advantage of BPT for patients who respond better to experiential rather than purely cognitive approaches. In both intervention groups, the changes in internal and external control beliefs were lower than expected; but when comparing the results with the reference sample, the findings in the IGs indicate an approximation of the normal range: Before treatment, the internal locus of control in all groups was below the normal range of the overall reference sample (4.12 ± 0.81), which is typical for people suffering from depressive disorders, while it was within the normal range in both IGs’ post-treatment. At the one-year follow-up, all three groups were in the lower normal range. The external locus of control was within the normal range in all groups at all assessment points (2.56 ± 0.96). Also contrary to our expectations, no significant differences were found in cognitive reflection (CRT-3) between groups. This finding suggests that improved cognitive processing might not be a primary mechanism in either intervention or that the CRT-3 might not adequately capture the specific cognitive changes targeted by these therapies.

These findings collectively indicate that while both WISE-T and BPT effectively reduce depressive symptoms, they operate through partially overlapping but distinct mechanisms. The effectiveness of WISE-T may be related to its focus on helping patients concentrate on what is truly important. By identifying important activities, scheduling them, and actively engaging in them, patients may establish a meaningful daily structure, engage in behavioral activation, enhance their sense of coherence, and interrupt rumination cycles. This approach may be particularly valuable in depression treatment, where cognitive overload can impede therapeutic progress. WISE-T appears to work primarily through cognitive pathways, whereas BPT combines cognitive benefits with unique body-focused mechanisms (physical mindfulness). This differentiation supports the value of offering multiple treatment options to address the heterogeneous nature of depression, allowing for personalized therapeutic approaches based on individual needs. The qualitative analysis of one-hour interviews with patients from the intervention groups, which has not yet been published, is intended to provide additional insight into the specific underlying mechanisms from the patients’ perspective.

The study has several notable strengths. It validates a novel therapeutic approach that was based on well-established principles from behavioral economics and cognitive science in a randomized, controlled, and naturalistic design, balancing methodological stringency with practical applicability. This design choice enhanced both internal validity (through the use of rigorous methodology) and external validity (through the real-world setting). The sample was heterogeneous in age, gender, and concurrent treatments, further supporting the study’s generalizability. The group setting offered several therapeutic and economic advantages.

### Limitations

Several limitations warrant consideration. First, the single-center design may limit generalizability of the findings, as treatment outcomes may be influenced by regional, social, temporal or cultural factors that differ from those present in the study setting. The sample consisted largely of participants with relatively high educational attainment and stable employment, which may limit generalizability to less well-resourced populations. Future studies should aim to include more diverse samples. Second, concurrent treatments were uncontrolled, leading to a heterogenous TAU condition [60]. 68% of the sample received specialized treatment against depression, and thus, the effect sizes were likely underestimated compared with a pure waitlist group [61]. Third, the sample size, while sufficient for moderate between-group effects, may have been too small for subgroup analyses. Fourth, therapist effects could not be fully separated from treatment effects.

## Conclusions

This randomized controlled trial demonstrates that both WISE-T and BPT are effective treatments for depression in this single-center setting. Deliverable in group formats, both therapies address the growing gap between mental health treatment demands and resources. WISE-T’s emphasis on identifying what is important, scheduling meaningful activities, and engaging in them introduces a novel therapeutic mechanism and complements existing treatments.

## Supplementary information

Additional file 1: Core Components of WISE-T. This figure illustrates the key elements and cyclical process of WISE-Therapy (WISE-T; What’s Important: Schedule and Engage). The therapy integrates four interconnected components: Values: Identifying and aligning with personal core values. Habits: Developing sustainable, positive behavioral patterns. Clear Thinking: Promoting rational descision making and cognitive clarity. Tools: Implementing practical strategies and techniques. The circular arrangement and arrows indicate the continuous, reinforcing nature of these components. By addressing each area, the therapy aims to enhance focus on important life aspects, fostering long-term well-being and effective life management for individuals with depression

Additional file 2: Overview of the WISE-T sessions

## Data Availability

The fully anonymized dataset supporting the conclusions of this article is available in Zenodo at 10.5281/zenodo.15211843 upon publication.

## Abbreviations

AE: Adverse Event
ANOVA: Analysis of Variance
BMI: Body Mass Index
BPT: Bouldering Psychotherapy
CBT: Cognitive-Behavioral Therapy
CG: Control Group
CI: Confidence Interval
CRT: Cognitive Reflection Test
EV: Explanatory Variables
GAD-7: Generalized Anxiety Disorder Scale-7
HMM: Hybrid Marginal Model
IE-4: Internal-External Locus of Control Scale
IG: Intervention Group
IMBE: Institute of Medical Informatics, Biometry, and Epidemiology
WISE-T: WISE-Therapy (What’s Important: Schedule and Engage)
LMM: Linear Mixed Model
LRT: Likelihood Ratio Test
MADRS: Montgomery-Asberg Depression Rating Scale
MAR: Missing At Random
MDD: Major Depressive Disorder
MM: Marginal Models
SD: Standard Deviation
SE: Standard Error
SIGMA: Structured Interview Guide for the Montgomery-Asberg Depression Rating Scale
SMS-PA-12: State Mindfulness Scale for Physical Activity
SMS-PA-G: German version of the SMS-PA
SOC-L9: short nine-item version of the Sense of Coherence Scale (SOC)
SWE: Self-Efficacy Scale (German: Skala zur Allgemeinen Selbstwirksamkeitserwartung)
TAU: Treatment as Usual
t0: pretest at baseline
t1: posttest after the intervention period
t2: follow-up after one year
