# Supplementary material for: WISE-Therapy (What’s Important: Schedule and Engage) and bouldering psychotherapy for depression: A randomized clinical trial
Source: BMC Med. 2026 May 27;24:332. doi: 10.1186/s12916-026-04918-5 (PMC13214313; doi:10.1186/s12916-026-04918-5)
Supplement: Supplementary file 1 — Additional file 1: Core Components of WISE-T. This figure illustrates the key elements and cyclical process of WISE-Therapy (WISE-T; What’s Important: Schedule and Engage). The therapy integrates four interconnected components: Values: Identifying and aligning with personal core values. Habits: Developing sustainable, positive behavioral patterns. Clear Thinking: Promoting rational descision making and cognitive clarity. Tools: Implementing practical strategies and techniques. The circular arrangement and arrows indicate the continuous, reinforcing nature of these components. By addressing each area, the therapy aims to enhance focus on important life aspects, fostering long-term well-being and effective life management for individuals with depression [file 12916_2026_4918_MOESM1_ESM.docx]

Clear Thinking

Values

Tools

Habits

WISE-T

**Additional file 1.** Core Components of WISE-T**.** This figure illustrates the key elements and cyclical process of the What’s Important: Schedule and Engage - Therapy (WISE-T). The therapy integrates four interconnected components: Values: Identifying and aligning with personal core values. Habits: Developing sustainable, positive behavioral patterns. Clear Thinking: Promoting rational decision making and cognitive clarity. Tools: Implementing practical strategies and techniques. The circular arrangement and arrows indicate the continuous, reinforcing nature of these components in WISE-T. By addressing each area, the therapy aims to enhance focus on important but non-urgent life aspects, fostering long-term well-being and effective life management for individuals with depression.
