# Supplementary material for: WISE-Therapy (What’s Important: Schedule and Engage) and bouldering psychotherapy for depression: A randomized clinical trial
Source: BMC Med. 2026 May 27;24:332. doi: 10.1186/s12916-026-04918-5 (PMC13214313; doi:10.1186/s12916-026-04918-5)
Supplement: Supplementary file 2 — Additional file 2: Overview of the WISE-T sessions [file 12916_2026_4918_MOESM2_ESM.docx]

**Additional file 2.** Overview of the WISE-T sessions

| **Session** | **Topic** | **Suggested Reading** | **Category** | **How It Promotes Focus on What Is Important** |
| --- | --- | --- | --- | --- |
| 1 | Eisenhower Matrix | 1, 2 | C | Helps patients identify and prioritize important but non-urgent activities |
|  | Value Clarification | 3 | V | Aligns important activities with personal core values |
| 2 | Habits | 4 | H | Encourages formation of habits supporting activities |
|  | Pareto Principle | 5 | C | Focuses attention on high-impact important activities |
| 3 | Balance Model | 2, 6 | C | Promotes understanding of life balance, encouraging engagement in important activities |
|  | Ivy-Lee Method | 7 | T | Provides practical tools for daily prioritization of important tasks |
| 4 | Solomon Paradox | 8 | C | Enhances objective self-reflection, promoting wiser decisions about important activities |
|  | Focusing Illusion | 9 | C | Helps identify truly important activities beyond immediate concerns |
| 5 | Saying No | 10 | T | Empowers patients to choose important activities over urgent demands |
|  | Clear Thinking | 11 | C | Provides frameworks for better decision-making about important activities |
| 6 | Active Language | 12-15 | H, C | Promotes active engagement in important activities through the use of language |
|  | Confirmation Bias | 16, 17 | C | Improves objective evaluation of important activities and their outcomes |
| 7 | Value Clarification | 3 | V | Aligns important activities with personal values and life purpose |
|  | Sunk Cost Fallacy | 18 | C | Reduces commitment to unproductive tasks, freeing time for important activities |
| 8 | Hindsight Bias | 19 | C | Improves learning from past experiences to better plan important activities |
|  | Decision-Making Strategies | 20 | T | Enhances decision-making process for important activities; decision-making as an important activity in itself |
| 9 | Radical Acceptance | 21 | C | Facilitates focus on changeable, important aspects of life |
|  | Occam's Razor | 22 | C | Simplifies problem-solving, making important activities more approachable |
| 10 | Reflection and Consolidation | 23, 24 | H, V, C, T | Consolidates learning; identifies successful strategies for engaging in important activities |
|  | Transfer to Everyday Life | 25 | H | Plans for continued focus on important activities post-therapy, developing long-term habits |

Sessions were held weekly, with each session consisting of two hours including a break. In the first session, each participant prioritized an important activity for themselves. At the end of each session, homework assignments were discussed, and a brief summary was provided. From the second session onwards, each session began with a review of the homework and discussion of the individually agreed upon regular new important activity established in Session 1, in order to facilitate the development of a positive habit. While sessions 1, 7, and 10 explicitly concentrated on personal values, the consistent emphasis on important activities throughout all sessions served as a continuous process of value clarification, repeatedly engaging participants with their core priorities.

Each session consisted of three phases: a 20-minute starting phase, one or two 85-minute active phases with a 10-minute break, and a 15-minute closing phase. The starting phase included a brief round in which participants shared their current state of mind and discussed challenges from the previous session’s homework. Participants initially divided into smaller, rotating groups to foster personal connections and facilitate idea exchange. During the active phase(s), groups reconvened to discuss session topics collectively before engaging in topic-related interventions, which sometimes involved teamwork or individual tasks aimed at addressing cognitive distortions. Sessions concluded with a closing phase in which participants received homework assignments and shared personal insights and key takeaways in a group discussion.

Abbreviations: C, Clear Thinking; H, Habits; T, Tools; V, Values.

**Suggested Reading**

1. Covey SR. The 7 habits of highly effective people. 30th ed: Simon & Schuster; 2020. | 2. Zhu M, Yang Y, Hsee CK. The mere urgency effect. Journal of Consumer Research. 2018;45(3):673-90. | 3. Cohen GL, Sherman DK. The psychology of change: self-affirmation and social psychological intervention. Annual Review of Psychology. 2014;65:333-71. | 4. Carden L, Wood W. Habit formation and change. Current Opinion in Behavioral Sciences. 2018;20:117-22. | 5. Koch R. The 80/20 Principle: Achieve More with Less. 4th ed: Nicholas Brealey Publishing; 2022. | 6. König CJ, Kleinmann M. Time management problems and discounted utility. The Journal of Psychology. 2007;141(3):321-34. | 7. Clear J. The Ivy Lee method: The daily routine experts recommend for peak productivity2020. Available from: <https://jamesclear.com/ivy-lee>. | 8. Grossmann I, Kross E. Exploring Solomon's paradox: Self-distancing eliminates the self-other asymmetry in wise reasoning about close relationships in younger and older adults. Psychological Science. 2014;25(8):1571-80. | 9. Kahneman D, Krueger AB, Schkade D, Schwarz N, Stone AA. Would you be happier if you were richer? A focusing illusion. Science. 2006;312(5782):1908-10. | 10. Hinton AO, McReynolds MR, Martinez D, Shuler HD, Termini CM. The power of saying no. EMBO reports. 2020;21(7):e50918. | 11. Kahneman D. Thinking Fast and Slow: Penguin; 2012. | 12. Bem DJ. Self-perception theory. In: Berkowitz L, editor. Advances in Experimental Social Psychology. 6: Academic Press; 1972. p. 1-62. | 13. Boroditsky L. How language shapes thought. Sci Am. 2011;304(2):62-5. | 14. Schacter DL, Buckner RL. Priming and the brain. Neuron. 1998;20(2):185-95. | 15. Kegan R, Lahey LL. How the way we talk can change the way we work: Seven languages for transformation: John Wiley & Sons; 2002. | 16. Kappes A, Harvey AH, Lohrenz T, Montague PR, Sharot T. Confirmation bias in the utilization of others’ opinion strength. Nature Neuroscience. 2020;23(1):130-7. | 17. Nickerson RS. Confirmation bias: A ubiquitous phenomenon in many guises. Review of General Psychology. 1998;2(2):175-220. | 18. Arkes HR, Blumer C. The psychology of sunk cost. Organizational Behavior and Human Decision Processes. 1985;35(1):124-40. | 19. Fischhoff B. Hindsight is not equal to foresight: The effect of outcome knowledge under judgment of uncertainty. J Exp Psychol Hum Percept Perform. 1975;1:288-99. | 20. Hammond JS, Keeney RL, Raiffa H. The hidden traps in decision making. Harvard business review. 1998;76(5):47-58. | 21. Aurelius M, Clay D, Hammond M. Meditations: Penguin Books Limited; 2006. | 22. Jefferys WH, Berger JO. Ockham's razor and Bayesian analysis. American scientist. 1992;80(1):64-72. | 23. Smith CD, Scarf D. Spacing repetitions over long timescales: A review and a reconsolidation explanation. Frontiers in Psychology. 2017;8. | 24. Tse D, Langston RF, Kakeyama M, Bethus I, Spooner PA, Wood ER, et al. Schemas and memory consolidation. Science. 2007;316(5821):76-82. | 25. Blume BD, Ford JK, Baldwin TT, Huang JL. Transfer of training: a meta-analytic review. Journal of Management. 2010;36(4):1065-105.
